# “Are You Doing Any Sport Science?” A Brief Editorial

**DOI:** 10.3390/jfmk7030069

**Published:** 2022-09-08

**Authors:** W. G. Hornsby, B. H. Gleason, M. DeLong, M. H. Stone

**Affiliations:** 1 School of Sport Sciences, College of Applied Human Sciences, West Virginia University, Morgantown, WV 26505, USA; 2 Department of Sport, Exercise, Recreation and Kinesiology, East Tennessee State University, Johnson City, TN 37614, USA; 3 Center of Excellence for Sport Science and Coach Education, East Tennessee State University, Johnson City, TN 37614, USA

**Keywords:** sport science, athlete monitoring, sport scientist

## Abstract

This brief opinion-based editorial addresses what the authors perceive to be a fundamental issue in the application of sport science, and these issues are reflected by the question “Are you doing any sport science?” As sport science has grown within the United States, organizational sport science budgets have grown, with increasing interest in developing various sport science initiatives. While it is indeed an exciting time for sport science, the authors suggest that, too often, sport science pursuits are driven by commercially available technologies and viewed as an “add-on” instead of pursuing an integrated systematic approach to informing the training process.

## 1. Introduction

This brief introductory editorial to the *Journal of Functional Morphology and Kinesiology*’s Special Issue “Applied Physiology and Performance #2” aims to address a current issue within the present landscape of applied sport science within the U.S. 

The field of “sport science” appears to be rapidly growing within U.S. collegiate, international, and professional sports [1,2]. Jobs that are expected to lead by data-informed (decision-making) efforts are no longer a unique occurrence within a given sporting organization; within certain sports leagues, they are now commonplace (e.g., MLB, NBA, MLS, NFL, etc. in the U.S.) or are beginning to become commonplace (e.g., NCAA) [1,2]. Often, these data pursuits focus on athlete assessments and athlete monitoring. In this editorial, our discussion targets training-process-focused applied sport science. Additionally, this discussion is based upon our U.S. experience.

## 2. The Growth of Sport Science within the U.S.

To our knowledge, prior to the late 2000s, the only truly full-time applied sport science positions within any U.S. sporting organization were at the U.S. Olympic Training Center (USOTC). Examples of a few of these sport scientists (all of whom worked at the Colorado Springs, CO, USA, USOTC) include Dr. Randy Wilbur (senior sport physiologist, 1993), Dr. Bill Sands (Director of Biomechanics and the USOTC Recovery Center, 2002), and Dr. Mike Stone (Director of Physiology, 2002). Several other countries (Australia, Germany, the United Kingdom, and Finland) also invested in sport science (jobs, training integration, research, etc.) through national- or state-level Olympic training centers prior to sport science becoming more popular at other sporting levels within their respective nations (e.g., professional teams hiring sport scientists and investing in instrumentation). 

While it is an exciting time for the profession, this apparent “sport science boom” is also an important time for the field to display the worth of a highly scientific approach to athlete development and performance to various stakeholders and management (e.g., NCAA athletic directors, professional sports general managers, etc.). Several articles have previously addressed systemic and structural issues that can make this complicated and potentially difficult. A few of these challenges include the following:

- A lack of mandatory sports coach education in advanced and high-performance sports [3,4];
- Individuals who are not really sport scientists calling their work “sport science” (i.e., data analysts, strength and conditioning coaches, etc.) [3,5];
- The widespread use of unvetted technology [5,6];
- Technology and devices driving the system rather than questions being asked and answers to problems being sought [5,6];
- The organizational structure of many sport organizations making integration and communication difficult (i.e., reporting hierarchy, job roles, etc.) [1,2,7,8,9];
- Supervisors of sport scientists and sport science initiatives rarely having a background in the area, leading to a poor understanding of what a sport science program can or should do [1,3];
- Sport scientists who are able to obtain full-time employment in that capacity must create, explain, or defend their job on their own [1].

In addition, limited opportunities exist for sport science to inform sports policy due to access and funding issues. Additionally, no professional standards exist for sport scientists.

## 3. A Concerning Question

A concerning question we often hear or receive that perhaps reflects the challenges listed above and that identifies a current sport science problem is “Are you doing any sport science?” Based on our experience, this question really aims to ask “What measurements do you make?” A typical response to this question lists the various assessments, measures, and monitoring strategies one (or a staff) uses. We believe that the specific language and framing of the question “Are you doing any sport science?” encapsulates how sport science is too often viewed as separate from or an add-on to the training process. Particularly concerning is how often the answer to this question is met with a list of commercially available devices and technologies and often neglects the specific measures and, most importantly, the goals and purpose(s) of the various assessments. 

We argue that training-process-driven sport science should be at the forefront of a high-performance model. This approach begins with (1) a diligently scripted, evidence-led plan; (2) high-level coaching and implementation; and (3) frequent monitoring at various “levels”, allowing the athlete’s development and performance to be guided. Within that process, the approach to assessments and monitoring heavily relies on the following:

- The stage and (biological and chronological) age of the athlete;
- The expertise of those involved;
- The available resources;
- Coach/athlete receptiveness.

Certainly, assessments aimed at answering specific questions that are somewhat removed from day-to-day training and coaching considerations also have an important place within sport science [10,11] (e.g., predicting what performance elements are necessary to win at big-time competitions, formulating and answering specific technical or tactical questions, optimizing talent ID programs, shaping league rules to improve athlete performance and to protect health, etc.). These topics are not simply “add-ons”, but rather relevant questions that can inform or improve the training process, potentially both for the athletes in question and other athletes as well. The integration and analysis of large datasets can be performed to guide sports rules and policies, integrating best practices to foster performance enhancement and injury reduction. This requires humans to collect and interpret the data.

**Figure 1:**
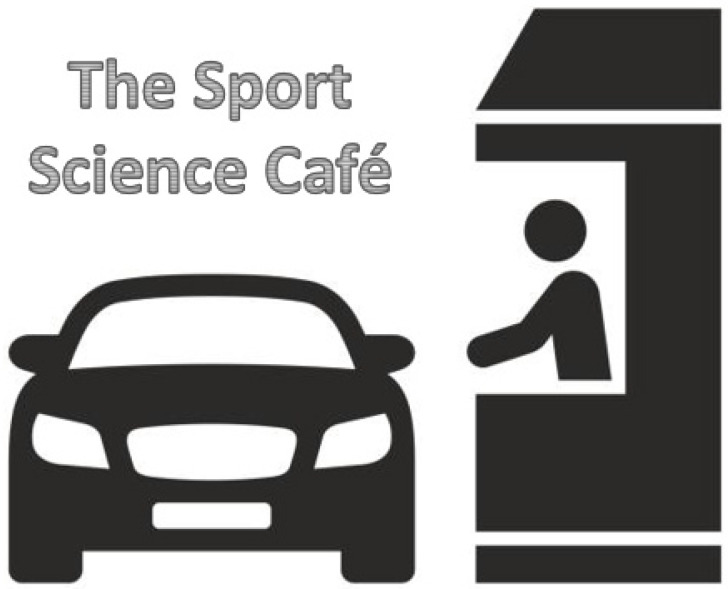
“The Sport Science Café”.

In the 2003 song *The Horizon Has Been Defeated,* musician Jack Johnson said, “no prints can come from fingers if machines become our hands.” It is our fear that technological development within sport science is leading to a seemingly cookie-cutter approach, in which a checklist or a menu-driven structure is the focal point (e.g., what technology is used). This is a clear diversion from people- and process-driven sport science approaches. Analogous to a fast food restaurant, sports organizations are pulling up to the *Sport Science Café* drive-through (Figure 1), selecting a few items off the menu, subsequently checking the “we’re doing sport science” box, and driving on.

To offer an additional analogy, some houses were built in the 1800s. These houses were constructed by hand, with the aid of tools. When they were completed, the houses effectively provided shelter, safety, and a home. Some of these houses still stand today, and many provide impressive reminders of quality processes: human creativity, ingenuity, beautiful design, quality workmanship, and attention to detail. At present, most of the tools with which these old houses were built (hammer, nails, etc.) still exist; however, the processes for constructing houses have evolved greatly over the past 200–250 years. There was nothing wrong with those 19th century tools; however, humans have developed technologies that increased the building speed to a pace that was not possible in the 1800s. In comparison, a large number of homes can now be built in planned housing developments very quickly. Despite these improvements in efficiency, one may argue that today’s rapidly built homes lack the individualization that previous methods offered, and some materials can be less sturdy than older methods. 

High-level coaches used scientific approaches in which measurements helped inform their coaching well before today’s current technological offerings. One outstanding example is swimming coach “Doc” Counsilman. 

## 4. Doc Counsilman

J. E. “Doc” Counsilman (1920–2004) epitomized the use of a scientific approach [12]. Doc was a competitive swimmer at Ohio State University, where he set world records for the 50- and 300-yard breaststrokes. Following his military service in WWII, Counsilman earned graduate degrees in physiology from both the University of Illinois and the University of Iowa while serving as an assistant swimming coach. He climbed the ranks in academia, from assistant professor to associate professor at Iowa State University and earned professorship at the State University of New York College at Cortland. In 1963, he became an assistant professor and the head swim coach at the University of Indiana, where he remained until his retirement in 1990. Counsilman produced stellar teams at the University of Indiana, winning several national championships, producing many elite swimmers and world-record breakers. In recognition of his coaching prowess, he was named an Olympic Coach in 1964 and 1976.

He was quite innovative and creative in combining science and sport. A few of his innovations include the following:

- In 1948, he pioneered the use of underwater photography in analyzing stroke mechanics.
- In 1949, he developed a protocol for the use of weight training in the conditioning of competitive swimmers.
- In 1949, he introduced interval training for competitive swimming and invented a pace clock that permitted the application of interval training routines specific to competitive swimming.
- In 1969, he used underwater photographic analysis to show the curvilinear pulling patterns of swimming strokes as well as the role of lift (Bernoulli effect) in the propulsion produced by these curved line sculling motions.
- In 1979, he pioneered the use of underwater photography for investigations into the acceleration phases in swimming.

As a tribute to Doc, the USOC created the “Doc” Counsilman Award. This annual award recognizes coaches who use scientific techniques and equipment as an integral part of their coaching methods or creates innovative ways to use sport science.

## 5. Devices and Instruments Are Tools for Sport Science, Not Science in and of Itself

Advancements in exercise and sport science have led to more automated and efficient processes. For example, a metabolic cart has replaced the Douglas bag; video software has replaced having to painstakingly assess pictures frame by frame for biomechanical analysis; and so on. We are very thankful for the continued advancements in instrumentation and technology as it has made our lives much easier, but we also recognize that more automated processes likely result in less “fingerprints”—perhaps, at times, less human interaction and less attention to detail. Thus, by relying too much on technology, the sport scientist may more easily ignore the nuances and subtleties of data collection, data handling, and data processing, and reduce the volume of interactions with those whom they are charged to help. 

A consideration for developing student sport scientists involves our typical instructional force plate laboratory activities that we teach (or have taught) at our respective universities. While we very much appreciate automated commercially available force plates with software designed to effectively and efficiently perform analyses of sport movements (e.g., jump and strength analyses), we believe the better learning experience entails the student collecting force data and analyzing the data in a non-automatic manner (e.g., using LabVIEW (NI, Austin, TX, USA) or MATLAB (MathWorks, Natick, MA, USA)). Using these programs, often the student must analyze each trial individually in a more “by hand” manner. While many university class-based labs likely still involve this scenario, we are concerned that students may be introduced to force plate technology via a sport science internship within the university’s athletic department and receive a less formal education and training experience that may emphasize speed over understanding. 

It is worth noting that the majority of popular commercially available sport science products are in some way related to an athlete’s physiology, biomechanical aspects, workload, fatigue, preparedness, etc. Thus, a need exists for those using these instruments to have a strong background in physiology or biomechanics as well as a sound understanding in basic research design and methodology (e.g., validity and reliability) [1,11]. For example, commonly used devices include the following:

- Force plates, or other devices containing force transducers, dynamometers, strain gauges, etc.;
- Barbell velocity devices;
- Global Positioning Systems (GPS);
- Devices for assessing heart rate and heart-rate variability;
- Devices for assessing sleep;
- Surveys obtaining subjective assessments of well-being, soreness, recovery, injury, etc., collected by a computer application or by pen and paper.

These instruments provide measurements that attempt to quantify aspects such as force (strength), explosive strength (rate of force development) and power, training workloads, fitness capabilities, and outside stressors. The list above is purposeful in that these are currently popular technology areas in which teams and athletic departments are spending money on, often at very high price points.

It is important to appreciate some of the more “established” and, perhaps, admittedly, boring measurements that are, for good reason, cornerstones of measurement in sport science. If the answer to the question “Are you doing any sport science?” was “Yes, we frequently measure hydration, body mass, load and reps performed, distance, and time run and periodically measure body composition, anthropometrics, sprint times, and jump heights”, some, sadly, may immediately view that as “They are not doing sport science” because these measurements are not the “trendy” go-to examples of sport science. Like the methods of the 1800s carpenter, there is nothing wrong with a simple sport science program if it suits the needs and budget of the sport organization.

## 6. Communicating Data 

Similar to the above commentary, providing graphs and charts—even fancy ones—does not equate to “doing” sport science. However, the ability of the sport scientist to communicate is undoubtedly an incredibly important skill. Often, the person leading sport science initiatives is not the person writing and leading the training prescription. A coach has an obligation and responsibility to interact with the sport scientist to enhance their own awareness of the local training process. Certainly, data visualization can be a helpful way to communicate data, but recently, we have observed that the use of athlete management systems is too often replacing relevant conversations with coaches and stakeholders. Managing, handling, and communicating data are core sport scientist challenges; establishing ways to better handle, share, and communicate data is not a new concept. Strength and conditioning coach Bob Ward created a reporting system with computer-driven analytics for the Dallas Cowboys all the way back in the mid-1970s. He and Dr. Ralph Mann also conducted on-field player tracking, a precursor to GPS. Pioneering sport scientist Dr. Bill Sands created and utilized aspects of an athlete management system (AMS) over 30 years ago, building computer-based reporting systems, writing code, creating AI capabilities, etc., years before commercial AMS options were available. Dr. Sands has written and presented on his system and its specific aspects many times over the years. 

AMS help in three key areas: (1) providing a place for various data streams to be stored, (2) providing tools to analyze information and to construct results (answers to relevant questions), and (3) communicating findings to stakeholders (coaches, sports medicine staff, nutrition staff, etc.). Like any tool, an AMS can be used poorly. An all too common scenario involves data from wearable devices streaming into an AMS via an application programming interface (API), and preset calculations and graphics being produced automatically such that the coaching staff can log in and view those calculations and graphics at their convenience without communicating with a sport scientist. Though this automated process may be largely motivated by budgetary limitations in many organizations (demonstrated by strength coaches using technology so that sports coaches can attract recruits), it lacks “fingerprints”—the context and meaning behind the results are largely absent and may not be received in the intended manner. 

In addition, we have personally observed that data reported in this way can be distracting to the coach’s training process. Some primary pitfalls of solely relying on an AMS and neglecting in-person communication and discussion include the following:

- The coach may not use the AMS.
- The coach may misinterpret the results presented or misunderstand their significance.
- The sport scientist may not provide relevant information to the coach.
- The coaching staff and athletes may view the sport scientist as an “outsider” when there comes a time to meet and discuss certain processes.
- When only working “within the AMS”, issues with data hygiene may be overlooked.

A common example within team sports is the use of AMS reports in which workload data (often GPS data) are framed in a context emphasizing competition to the coach. This is perceived by the sport coach by the philosophy “more work = better”. Sadly, sometimes in this scenario, a less fit or a less efficient athlete is applauded for working at a high exertion, but a fitter or more efficient (often more skilled) athlete is scolded for working at a lower level of overall exertion. In this scenario, the context of the game’s situations and the opportunities afforded are misinterpreted. For example, does the athlete have a reason to work hard or does the athlete wait patiently for a good opportunity within tactical constraints, and then quickly select and pursue a meaningful action with vigor? Additionally, tactical (soccer midfielder vs. defender) [13] and positional differences exist (basketball center vs. guard) [14] in each sport that must not be overlooked. This example demonstrates the importance of context-specific knowledge and communication that comprise the human element of delivering sport science-related information to coaches; it is not intended to suggest that an AMS is not helpful. On the contrary, advancements in AMS technologies have been one of the more exciting sport science developments in the recent decade. Borrowing Buchheit’s phrase here is appropriate: “Content is king, but context is God”. See https://hiitscience.com/content-is-king-but-context-is-god/ (accessed on 10 August 2022).

Perhaps *AMS reporting goes wrong* when technology is used in an effort to “do sport science”, and then the reporting is carried out in a manner only to show recruits, fans, the coach, and the administration that “we are doing sport science”. Sport science is applied to achieve a goal, so what then is the goal? The goal is none other than to produce recruitment videos, not to carry out sport science!

## 7. Case-Based Applied Sport Science

It is important for sport scientists and those making measurement-based decisions to appreciate the subtlety and nuances of carefully collected sport science data. A classic example, provided over the years by Dr. Bill Sands, Dr. Mike Stone, and others, is that, if 20 athletes undergo a training program, 17 of the athletes improve substantially, 1 athlete demonstrates trivial changes, and 2 athletes perform worse. A traditional analysis may show statistically significant alterations and may excitedly move forward to report the results to coaches (and/or submit to a journal). What if the two athletes who performed worse were the two Olympic hopefuls of the group? Their coach would likely consider the training program a failure. To resolve this issue and to apply “fingerprints” to the situation, a sport scientist may choose single-subject techniques to analyze the data and to provide appropriate context. These methods have long been an important topic of interest in applied sport science, as have the challenges of mean group data. Some important considerations include the following [15]:

- Athlete data from teams almost always involve a biased (not randomly assigned) sample;
- Athlete data are seldom normally distributed;
- Athletes have differing genetic profiles, training backgrounds, and experiences that are often much more pronounced between athletes; these may be inconsistent with the backgrounds of subjects from the general population;
- Trained athletes are closer to their genetic ceiling and, thus, can experience less noticeable (but still very important) adaptations;
- Smaller gains can be overshadowed by outside stressors, effort, coaching, environment, etc.;
- The same training program, especially a more “mixed methods” training program, performed multiple times by the same athlete can produce differing results;
- Challenges exist with classifying the levels of an athlete, as it pertains to both adaptation as well as performance level within their sport.

Thus, the idea of single-subject, case-based approaches is widely accepted in applied sport science, particularly in terms of day-in and day-out training environments (i.e., at the micro-level) [16]. However, even with this approach, the unpredictable nature of sport does not afford us the ability to predict specific outcomes. It does, however, (along with a detailed training plan) improve our qualitative (or characteristic) prediction and our ability to direct desired future adaptations as well as to manipulate preparedness [17,18,19]. This process requires “fingerprints”.

## 8. Final Thoughts

Simply making measurements does not equal sport science, but sport science cannot exist without measurements. Measurements are a critical initial step; however, just as important is *what the measurement is used for.* This is not to suggest that all data should be constantly immediately acted upon. A helpful process for assigning “immediacy” is by categorizing three monitoring buckets reflecting different time frames: (1) fatigue management (short-term); (2) program efficacy (medium-term); and (3) long-term projects such as collecting norms, calculating development trajectories for talent ID and development purposes, or adapting league rules due to perceived needs. Sport science is the integration of planning, coaching, training, and measurement underpinned by an understanding and appreciation of both sport and science. Sport science is a human process and should not be completely reliant upon technology; a major aspect is integrating the scientific process into the athletes’ training processes. This is carried out by sharing ideas and observations with others and, thus, influencing the athlete to enhance elements of their training process. Automated processes involving technology, machine learning, algorithms, etc., can be useful tools used to influence processes, but it takes humans to guide athletes and to design or re-engineer processes when analyses suggest that there is a problem. Many follow-up investigations may be required to solve a given problem, which often requires resources and relies more on well-trained humans than technology. Sport science requires a problem-solving process—a willingness to provide and accept a solution within the organization; it is not empty data collection carried out for publicity.
